# D-Dimer as a Novel Factor for Recurrent Chronic Subdural Hematoma: Development and Assessment of a New Predictive Nomogram

**DOI:** 10.3390/brainsci16101099

**Published:** 2026-10-12

**Authors:** Jiajun Wei, Zhiyang Li, Wenju Wang, Qiang Cai

**Affiliations:** 1 Department of Neurosurgery, Renmin Hospital of Wuhan University, No. 238, Jiefang Road, Wuchang District, Wuhan 430060, China; jiajunwei@whu.edu.cn (J.W.); 2018283020174@whu.edu.cn (Z.L.); 2 Department of Neurosurgery, Xiantao First People’s Hospital Affiliated to Changjiang University, Xiantao 433000, China

**Keywords:** chronic subdural hematoma, recurrence, predictive nomogram, postoperative, D-dimer

## Abstract

**Highlights:**

**What are the main findings?**

- D-dimer was identified as an independent novel risk factor for postoperative recurrence after chronic subdural hematoma surgery.
- The constructed nomogram achieved good discrimination, calibration and clinical benefit validated by internal bootstrap resampling and an external cohort.

**What are the implications of the main findings?**

- This prediction model enables individualized preoperative risk stratification for CSDH recurrence and assists clinical decision-making.
- The coagulation-inflammation pathway may underlie the association between D-dimer and CSDH recurrence.

**Abstract:**

Background: Chronic subdural hematoma (CSDH) is the most common bleeding disorder in the nervous system and has a high recurrence rate, posing a serious threat to the lives of elderly individuals. Early identification and adjustment of the treatment regimen in the perioperative period is crucial. The aim of this study was to create a perioperative predictive nomogram for the early prediction of recurrent CSDH (R-CSDH). Methods: A predictive model was developed including 632 patients with CSDH hospitalized between 1 January 2021 and 31 December 2025 from Wuhan University People’s Hospital. The 206 patients from the First People’s Hospital of Xiantao City who were hospitalized between 1 January 2022 and 31 December 2025 formed an external validation cohort. Minimum absolute shrinkage was used to optimize feature selection, and logistic regression analysis was applied to further select features and construct a risk prediction model for recurrent CSDH. The calibration, discrimination, and clinical utility of the predictive models were assessed using calibration plots, the C-index, the area under the curve (AUC), and decision curve analysis. External validation was performed by an external validation cohort. Findings: Age, CT hematoma size, hematoma location, atrophy, density, surgical procedure and D-dimer were the predictors for constructing the predictive nomogram, and the results showed that a logistic regression model based on the above seven factors had good calibration and discriminative ability (C-index: 0.925; 95% confidence interval: 0.893–0.957). The AUC of this predictive model was 0.92487. The calibration curve of the nomogram was close to the ideal diagonal. In addition, the decision curve analysis showed that the net benefit of the model was significantly better. External validation demonstrated the reliability of the predictive nomogram. Interpretation: D-dimer was found to be a novel factor for recurrent chronic subdural hematoma. Our study provides clinicians with an accurate tool for identification and early intervention and therapy to reduce the incidence of R-CSDH.

## 1. Introduction

Chronic subdural hematoma (CSDH) is one of the most common bleeding disorders in the nervous system, accounting for approximately 10% of all intracranial bleeding disorders [1]. It is commonly seen in elderly male patients, with an incidence rate of 80.1/100,000/year in the elderly population over 65 years of age, increasing to 127.1/100,000/year in the population over 80 years of age, and its overall incidence rate shows an increasing trend year by year [2,3]. The main treatment for symptomatic CSDH in clinical practice is surgery, and 5% to 33% of patients experience recurrence after surgery, requiring reoperation or even resulting in death. The mechanism of postoperative recurrence of CSDH is complex and unclear [4].

To date, there are few reports on the correlation between perioperative factors and CSDH recurrence, and no risk prediction model for perioperative factors has been developed to predict the occurrence of CSDH recurrence. As simple statistical visualization tools, nomograms have been widely adopted to estimate disease onset, progression, prognosis and survival outcomes in recent years [5]. Although abundant neurosurgical literature has addressed chronic subdural hematoma, reliable preoperative predictive tools integrating plasma D-dimer for recurrence-risk stratification are still scarce, since earlier prediction models frequently overlooked the utility of coagulation-related laboratory markers and thus limited their real-world application in routine clinical practice. Therefore, in this retrospective study, we aimed to construct and externally validate a nomogram combining D-dimer with multi-dimensional clinical-imaging variables for preoperative individual recurrence-risk assessment; our initial study design incorporated baseline demographic information, imaging features and laboratory test parameters, which improved predictive accuracy and endowed the model with considerable clinical practical value.

## 2. Methods

### 2.1. Study Design and Population

The aim of this study was to retrospectively investigate the data of patients with chronic subdural hematoma admitted to the People’s Hospital of Wuhan University and the First People’s Hospital of Xiantao City. The study protocol was approved by the Ethics Committee of the People’s Hospital of Wuhan University (WDRM2025-KS002) and the Ethics Committee of the First People’s Hospital of Xiantao (XTDYRM2025N06), and informed consent was waived.

In the training cohort, this retrospective study included 632 patients with chronic subdural hematoma who were hospitalized from 1 January 2021 to 31 December 2025 at Wuhan University People’s Hospital in China. In the validation cohort, from 1 January 2022 to 31 December 2025, 206 patients with chronic subdural hematoma were hospitalized in the First People’s Hospital of Xiantao City, China. The inclusion criteria were as follows: ① patients with symptoms of chronic intracranial hypertension and ② patients with head CT or MRI showing subdural hemorrhage or effusion. The exclusion criteria were as follows: ① subacute subdural hematoma; ② severe underlying diseases; ③ admission GCS score <5 or pupil dilatation (one or both sides); ④ conservative drug treatment; and ⑤ incomplete follow-up data.

### 2.2. Data Collection

Data collected from the profiles of the patients with chronic subdural hematoma included the patients’ general condition (sex, age, smoking, drinking, GCS score), past medical history (history of hypertension, previous craniotomy), medication history (antiplatelet treatment, history of head trauma), imaging (CT hematoma size, midline shift, hematoma location, atrophy, density), surgical procedure and risk factors associated with test results (platelet, albumin, D-dimer, INR).

### 2.3. Definitions

The patients presented again with clinical symptoms after surgery, and CSDH reaccumulation in the same area on the same side of the head was confirmed on CT or MRI, with the earliest recurrence occurring 1 week after surgery and the latest recurrence occurring at 3 months after surgery [6,7].

Radiologically, cerebral atrophy was defined on CT as widening of the cortical sulci and enlargement of the ventricular system relative to age-matched normative references. Hematoma density patterns (homogeneous, mixed, membranous, separated) were categorized according to previously published CT-based classification criteria for chronic subdural hematoma [8].

### 2.4. Statistical Analysis

All data, such as general condition, past medical history, drug history, surgical procedure, imaging information and test results, were expressed as counts (%). Statistical analysis was performed using R software (version 3.6.3; https://www.R-project.org, accessed on 17 February 2022). To reduce the dimensionality of the data, all factors were included in the least absolute shrinkage and selection operator (LASSO) method, which is suitable for selecting the best predictive features of R-CSDH risk. A total of 17 features with nonzero coefficients were selected in the LASSO regression model, and predictive models were developed using single- and multifactor logistic regression analyses. These factors were considered to have an odds ratio (OR) with a 95% CI and a *p* value. Variables selected by the LASSO regression were further assessed by univariate and multivariate logistic regression. All whole categorical variables retained in the LASSO model were incorporated into the final nomogram, even if some subcategories presented *p*-values > 0.05 in multivariate analysis. Calibration curves were plotted to assess the calibration of the R-CSDH nomogram. To quantify the discriminatory performance of the R-CSDH nomogram, the C-index was measured. In addition, the area under the curve (AUC) was calculated to quantify the predictive power of the nomogram. The R-CSDH nomogram was validated by bootstrap validation (1000 bootstrap resamples) to calculate the relative corrected C-index. External validation was performed using an external validation cohort to calculate the C-index. Decision curve analyses were performed to quantify net returns at different threshold probabilities, which allows testing the clinical utility of the R-CSDH nomogram.

## 3. Results

### 3.1. Baseline Characteristics of Training and External Validation Cohorts

**Figure 1:**
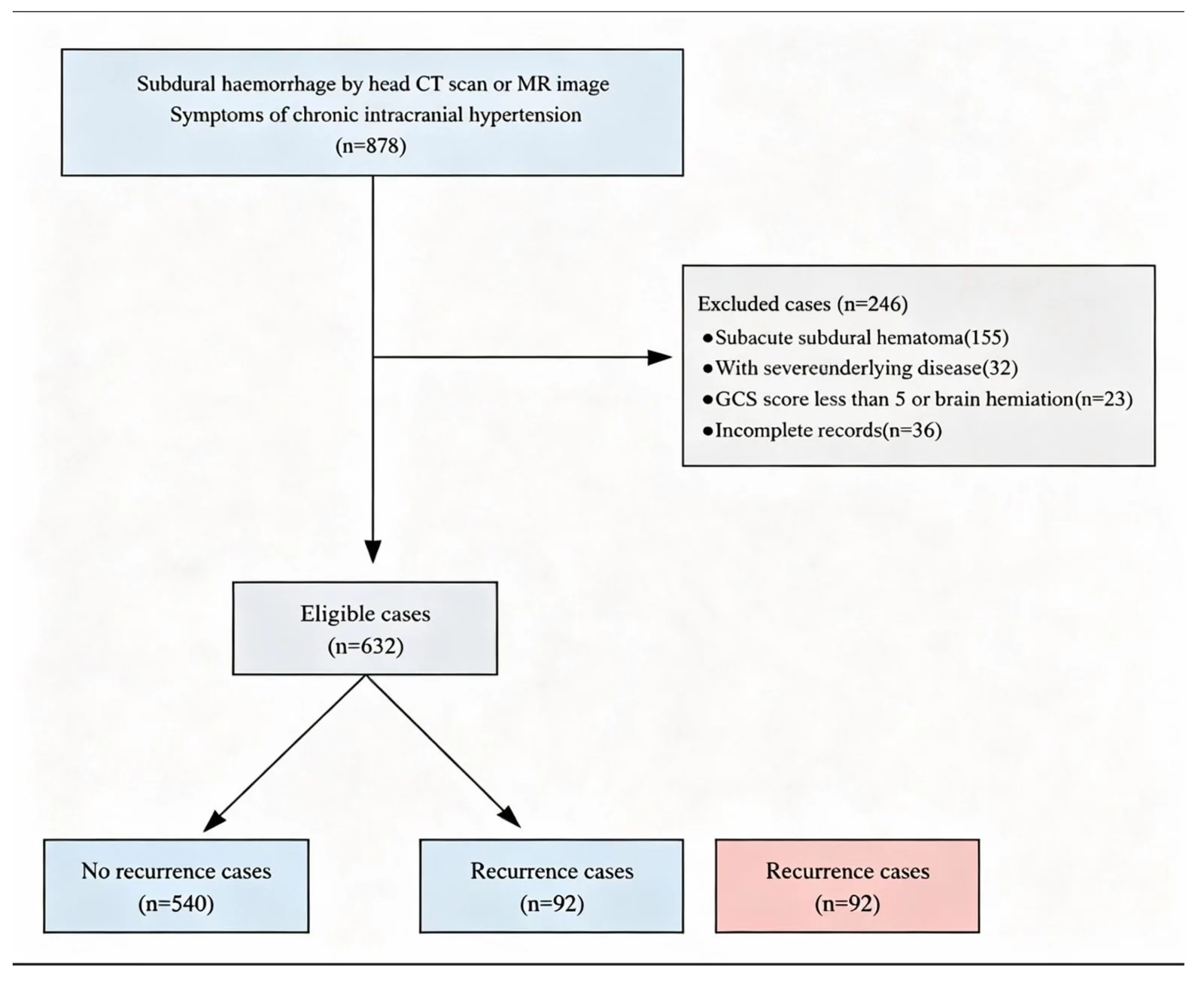
Flowchart illustrating patient screening and study cohort enrolment. A total of 878 patients with suspected chronic subdural hematoma (CSDH) were initially screened. Exclusion criteria included subacute subdural hematoma, severe underlying comorbidities, GCS score < 5, brain herniation, and incomplete follow-up records. Note that several patients met more than one exclusion criterion. Ultimately, 632 eligible patients were enrolled in the training cohort for nomogram construction, including 540 non-recurrence cases and 92 postoperative recurrence cases. Abbreviations: CSDH, chronic subdural hematoma; GCS, Glasgow Coma Scale.

In the training cohort, clinical information was collected from a total of 878 patients at the People’s Hospital of Wuhan University; 246 did not meet the inclusion criteria; 155 of the 246 had a diagnosis of subacute subdural hematoma; 32 suffered from a serious underlying disease; 23 had a GCS score of less than 5; and 36 had incomplete records, which meant that numerous items were missing in the collected data, including imaging information and postoperative follow-up. (Note: Some patients met more than one exclusion criterion.) Finally, 632 patients were included in this study (Figure 1). According to the calculations performed in PASS version 11.0.7, the minimum sample size was 148 (a = 0.05, power [1-b] = 0.9, and R2 of the predictive model was 0.15), and in this study, data from 632 CSDH patients at the People’s Hospital of Wuhan University were used to construct a nomogram, which was able to meet the sample size requirement. The percentages of patients aged less than 70 years, 70–80 years, and more than 80 years were 58.7%, 26.3%, and 15%, respectively, and the proportions of patients with hematoma volumes less than 100 cm^3^, 100–160 cm^3^, and more than 160 cm^3^ were 77.7%, 15.7%, and 6.6%, respectively. Overall, 22.9% of the patients had bilateral hematomas, and the CTs of 22.5% of the patients showed cerebral atrophy. The hematomas were classified according to their density as homogeneous, mixed, membranous, and separated in 21.2%, 43.5%, 12.8%, and 22.5% of the patients, respectively. A total of 55.2% of the patients underwent burr hole craniostomy, 31.5% of the patients underwent neuroendoscopy, and 13.3% of the patients underwent craniotomy. Plasma D-dimer was categorized into three groups of <0.5 mg/L, 0.5–2 mg/L and >2 mg/L, which accounted for 78.6%, 14.2% and 7.1% of the patients, respectively.

In the validation cohort, 206 patients from Xiantao People’s Hospital were used for external validation purposes. The proportions of patients aged less than 70 years, 70–80 years, and more than 80 years were 54.4%, 29.6%, and 16%, respectively, and the proportions of patients with hematoma volumes less than 100 cm^3^, 100–160 cm^3^, and more than 160 cm^3^ were 75.7%, 17.5%, and 6.8%, respectively. Overall, 27.2% of the patients had bilateral hematomas, and 20.4% of the patients had CT scans showing cerebral atrophy. The hematomas were categorized according to their density as homogeneous, mixed, membranous, and separated in 19.4%, 43.2%, 9.2%, and 28.2% of the patients, respectively. A total of 52.9% of the patients underwent burr hole craniostomy, 34% of the patients underwent neuroendoscopy, and 13.1% of the patients underwent craniotomy. Plasma D-dimer was categorized into three groups of <0.5 mg/L, 0.5–2 mg/L and >2 mg/L, which accounted for 77.2%, 16% and 6.8% of the patients, respectively.

According to the definition of R-CSDH patients, 92 patients (92/632, 14.6%) hospitalized in Wuhan University People’s Hospital were included in the relapse group, and 540 patients (540/632, 85.4%) were included in the nonrelapse group. There were no significant differences in sex or age between the two groups, and 21 clinical characteristics are listed (Table 1).

#### Feature Selection for Predictors of R-CSDH

**Figure 2:**
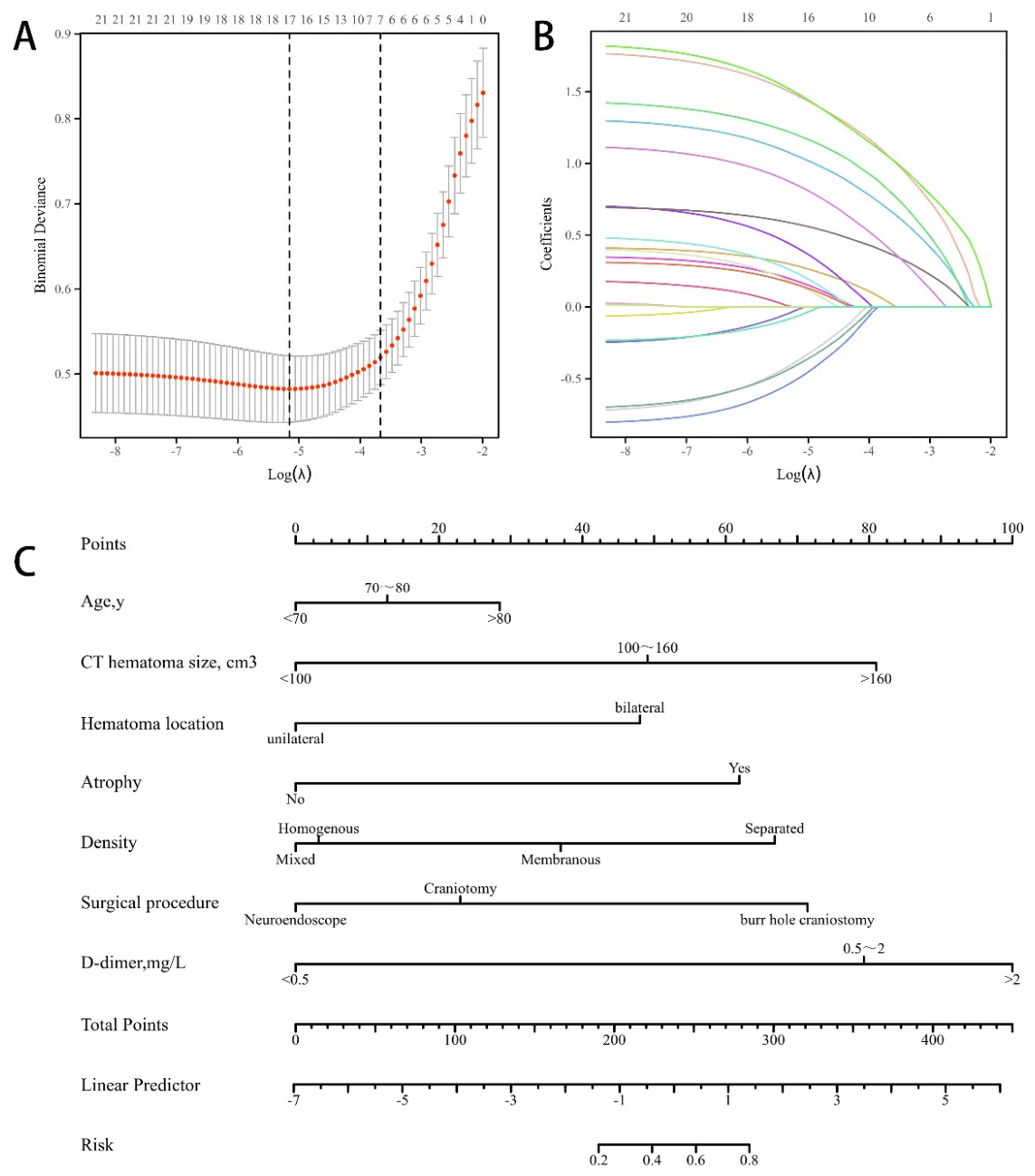
LASSO binary logistic regression model and the predictive nomogram for the risk of R-CSDH. (**A**) LASSO’s maximum parameter (lambda) was selected using fivefold cross-validation. To obtain the factor values, we used a partial likelihood deviance (binomial deviance) curve and a log (lambda) plot; (**B**) LASSO coefficient profiles of the 17 features. Based on the log (lambda) sequence, a coefficient profile plot was generated. We used fivefold cross-validation to select 17 features with non-zero coefficients and used LASSO, least absolute shrinkage, to draw a vertical line at these values. (**C**) A predictive nomogram was developed in the cohort based on age, CT hematoma size, hematoma location, atrophy, density, surgical procedure, and D-dimer.

Among the general condition, past medical history, medication history, and surgical procedure factors, as well as the imaging data and test results, 21 factors were reduced to 17 potential predictors based on the 632 patients with CSDH in the cohort (Figure 2A,B) that had nonzero coefficients in the LASSO regression model. These characteristics included age, smoking, drinking, history of hypertension, history of diabetes, anticoagulation treatment, antiplatelet treatment, history of head trauma, CT hematoma size (cm^3^), hematoma location, atrophy, density, GCS, surgical procedure, albumin, D-dimer and INR (Table 2).

### 3.2. Construction of the R-CSDH Predictive Nomogram

The results of the multivariate and univariate logistic regression analyses of the previously selected characteristics are shown in Table 2. The model incorporated predictors that were significantly different in the two analyses (*p* < 0.05), including age, CT hematoma size, hematoma location, atrophy, density, surgical procedure and D-dimer, and logistic regression models were constructed based on these seven factors. These seven factors in the logistic regression model were then integrated into the nomogram (R2 = 0.268, Cindex = 0.925) (Figure 2). The prediction equation created for the nomogram was:

LogitP= −2.5799 + [(0.4043*Age, 70~80) or (0.4043*Age, >80)] + [ (1.4637*CT hematoma size, 100~160) or (2.3978*CT hematoma size, >160)] + 1.4307*bilateral + 1.8299*Atrophy + [ (0.8368*Separated) or (−1.0863*Mixed) or (−0.9668*Homogeneous)] + [(−0.7235*Neuroendoscope) or (1.3931*burr hole craniostomy)] + [(−0.5812*D-dimer,0.5~2) or (−2.9304*D-dimer, <0.5)]. This predictive model is suitable for predicting the risk of postoperative recurrence in patients with chronic subdural hematomas based on current clinical features. Each predictive feature in the nomogram is assigned a segment of varying length. The length of the line segment indicates the importance of the feature. In other words, if a patient with chronic subdural hematoma has the clinical features listed in the nomogram before surgery, this indicates that the patient has an increased risk of postoperative recurrence.

### 3.3. Model Discrimination, Calibration and Clinical Net Benefit

**Figure 3:**
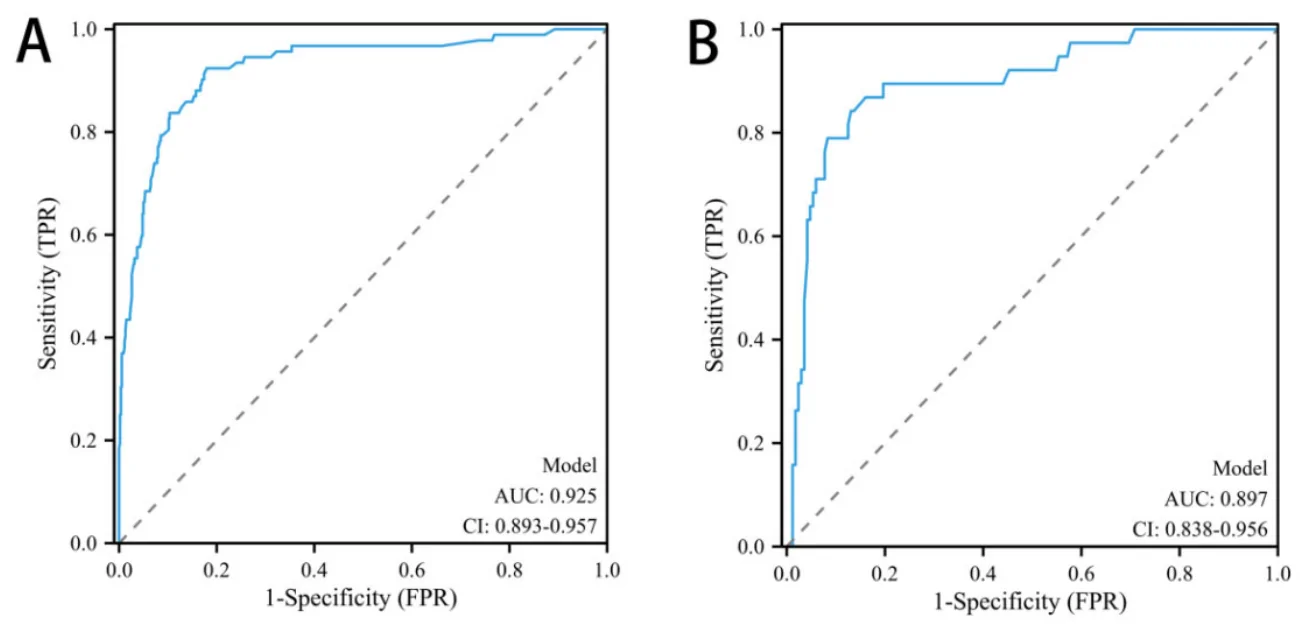
Receiver operating characteristic (ROC) curves of the nomogram for predicting recurrent chronic subdural hematoma (R-CSDH). (**A**) Training cohort (*n* = 632), AUC = 0.925, 95% CI: 0.893–0.957. (**B**) External validation cohort (*n* = 206), AUC = 0.897, 95% CI: 0.838–0.956. *X*-axis: 1-Specificity (false-positive rate); *Y*-axis: Sensitivity (true-positive rate). The dashed diagonal line represents random classification. Abbreviations: ROC, receiver operating characteristic; AUC, area under the curve; CI, confidence interval; R-CSDH, recurrent chronic subdural hematoma.

**Figure 4:**
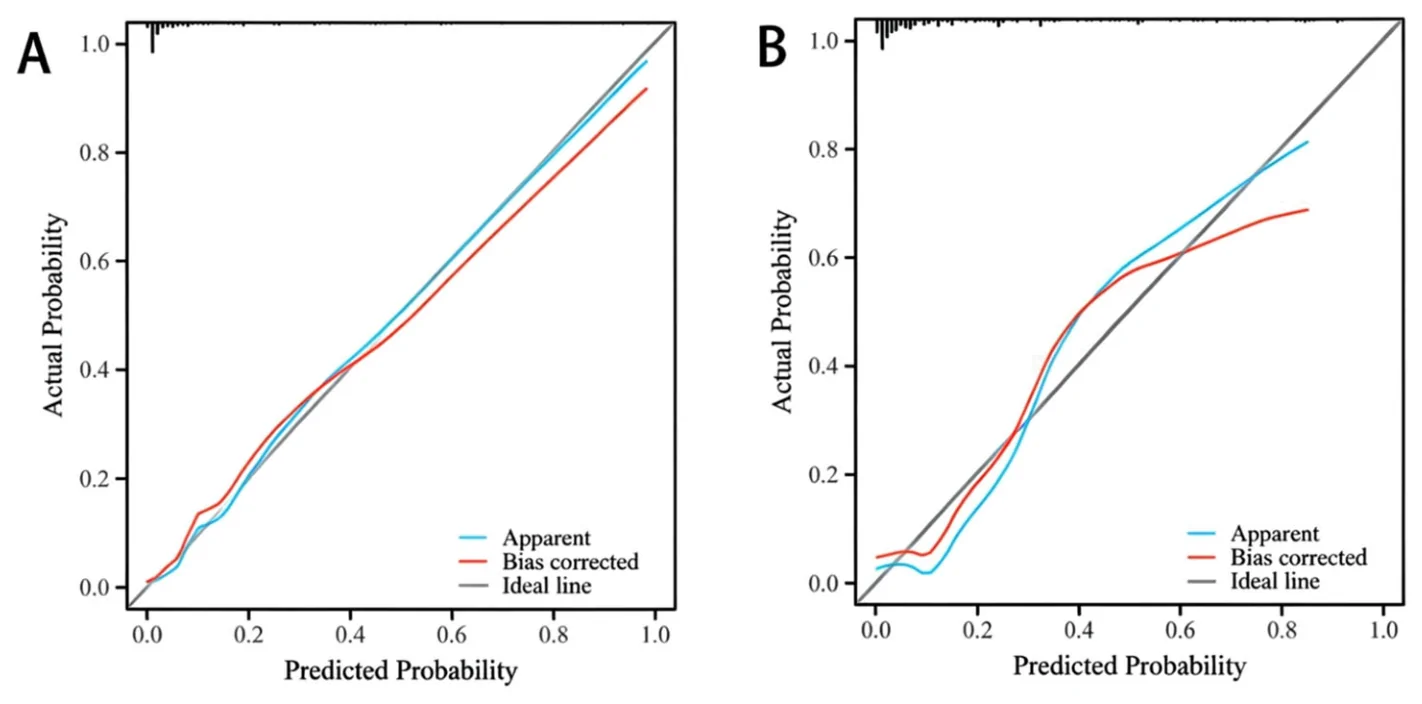
Calibration curves of the nomogram for predicting postoperative R-CSDH. (**A**) Training cohort (*n* = 632); (**B**) External validation cohort (*n* = 206). *X*-axis: nomogram-predicted recurrence probability; *Y*-axis: observed actual recurrence probability. Grey dashed line: ideal calibration. Blue line: apparent calibration; red line: bias-corrected calibration via 1000-bootstrap resampling. Abbreviation: R-CSDH, recurrent chronic subdural hematoma.

**Figure 5:**
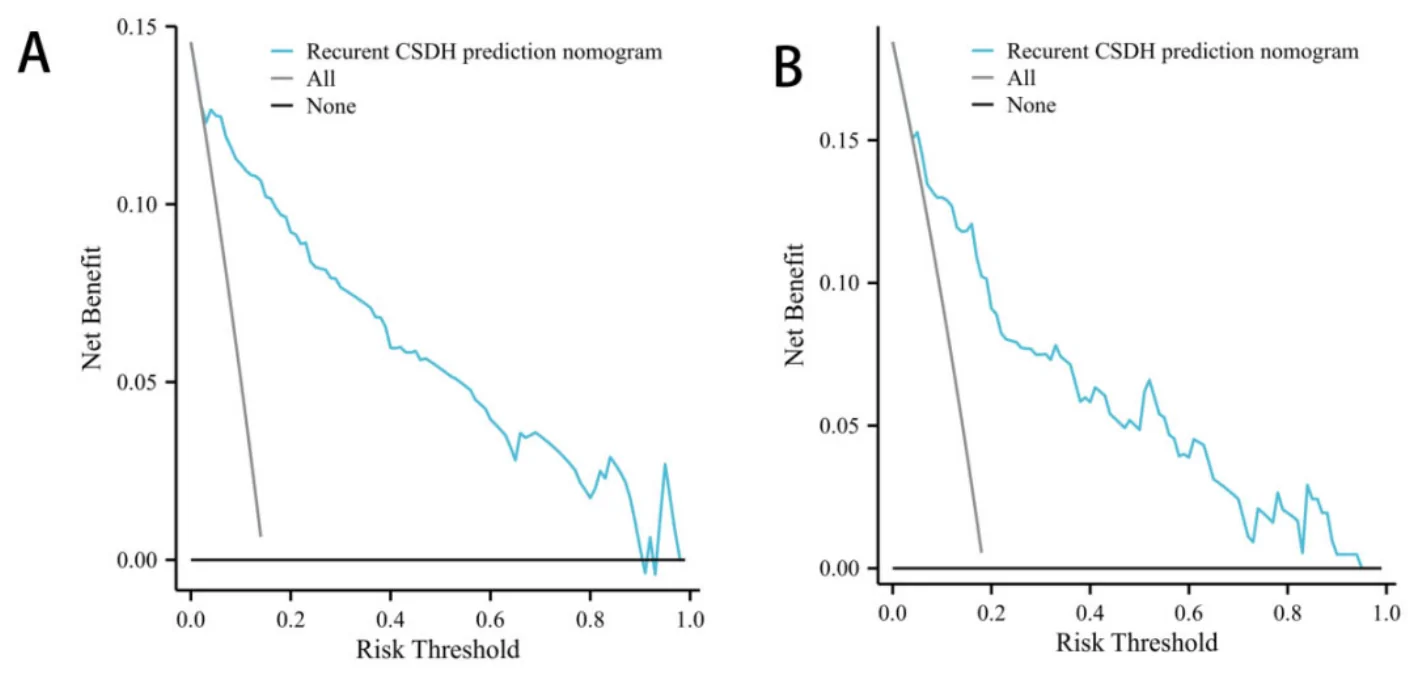
Decision-curve analysis (DCA) of the R-CSDH prediction nomogram. (**A**) Training cohort (*n* = 632); (**B**) External validation cohort (*n* = 206). *X*-axis: risk threshold probability; *Y*-axis: clinical net benefit. Cyan curve: nomogram; grey line: treat-all strategy; black line: treat-none strategy. Abbreviations: DCA, decision-curve analysis; R-CSDH, recurrent chronic subdural hematoma.

In the training cohort, the AUC was 0.925 (Figure 3A), and the calibration curve was close to the ideal diagonal (Figure 4A). In addition, DCA showed a significantly better net benefit for the predictive model (Figure 5A).

In addition, 206 patients from Xiantao People’s Hospital were used for external validation of the nomogram. The AUC was 0.897 (Figure 3B), indicating that the nomogram had not only good accuracy but also good consistency. The validation cohort calibration curve was also close to the ideal diagonal (Figure 4B). In addition, DCA showed a significant net benefit for both the predictive model and the validation cohort (Figure 5B). These data suggest that our nomograms have great potential for clinical decision-making.

## 4. Discussion

CSDH is a common disease in neurosurgery, and the main treatment for CSDH in clinical practice is surgery. Some patients may experience recurrence after surgery. Weigel et al. found that the recurrence rate was 33% in the craniotomy group, 12% in the drilling group, and 10.8% in the cranial group and that the overall recurrence rate of CSDH was between 5% and 33% [9]. The reasons for the recurrence of CSDH patients after surgery are complex, the mechanisms are not yet known, and the study of recurrence cases is necessary in clinical work. Currently, there is a lack of quantitative studies on the factors of postoperative recurrence in patients.

Many recurrence factors have been reported, such as advanced age and cerebral atrophy, which are independent risk factors for the recurrence of CSDH [10,11]. Chronic subdural hematoma represents a growing global health burden among elderly populations. Population ageing worldwide contributes to its continuously rising incidence, with reported incidence rates reaching 80.1 per 100,000 person-years in patients over 65 years old and even higher among octogenarians. Patients of advanced age themselves have varying degrees of cerebral atrophy and a history of cavernous cerebral infarction, and postoperative patients’ brain tissues may be poorly retracted, with impaired drainage of residual hematomas and effusions [10,12,13]. In addition, due to the increased mobility of brain tissue in the cranial cavity after cerebral atrophy, patients with minor head trauma after discharge from the hospital can develop a tear in the bridging veins and bleed again [14,15]. Current pathophysiological understanding suggests that CSDH postoperative recurrence cannot be simply attributed to residual hematoma volume alone. Instead, it arises from multifactorial crosstalk among age-related cerebral atrophy with impaired brain re-expansion, increased mechanical stretching and fragility of bridging veins, formation of vascularized hematoma inner–outer membranes, local inflammatory–angiogenic responses within these membranes, and repeated micro-oozing from fragile, newly formed neovasculature [16]. In this study, age > 80 years (OR = 2.475, 95% CI: 1.078–5.682, *p* = 0.033) and cerebral atrophy (OR = 6.234, 95% CI: 3.214–12.091, *p* < 0.001) indicated that age is positively correlated with the recurrence of CSDH, and as the patient’s age increases, the chance of postoperative recurrence increases. In a retrospective study, Myung-Hoon et al. [17] analyzed the clinical data of 756 patients with CSDH who underwent drilling and drainage surgery. A total of 104 (13.8%) patients with CSDH experienced postoperative recurrence during their 6-month follow-up, and the independent risk factors were found to be age >75 years, obesity (body mass index ≥ 25.0 kg/m^2^), and bilateral hematomas. During autopsies and microscopic evaluation of tissues from CSDH patients whose bridging vein connected to the dural portion of the vessel was thin, Yang AI et al. [18] showed that the patients had a low dural collagen content and that the dura was even more brittle than the arachnoid portion of the vessel, which was strongly associated with postoperative recurrence of CSDH. Older and R-CSDH patients had poorer mRS prognostic grading at 3 months, demonstrating that advanced age is not only an independent risk factor for postoperative recurrence in patients with CSDH but also indirectly affects the prognosis of patients because of the underlying diseases that often accompanies advanced age in patients with multiple recurrences of CSDH, leading to a poor prognosis. This is more consistent with the results of this study.

In this study, neuroendoscopic surgery for CSDH had a lower recurrence rate than drilling and drainage surgery. The reasons for this are summarized as follows: (1) neuroendoscopy itself has sufficient light and a clear field of view and can be used for close observation from multiple angles; (2) the presence of a fibrous septum in the hematoma will lead to poor drainage of drilling surgery alone, while with neuroendoscopic surgery, the hematoma can be removed under direct vision, the fibrous septum can be opened up, and the rate of hematoma removal can be increased; (3) the operator can peel off and remove the outer membrane of the hematoma as much as possible under the vision of the neuroendoscope, which results in a low recurrence rate; (4) the incision for neuroendoscopic surgery only needs to be 4–5 cm long, and the flap of the bone is approximately 3 cm, which provides a better balance between minimal invasiveness and effectiveness; and (5) a drainage tube can be placed under the guidance of endoscopy, and the accuracy of its placement can be ensured [19,20].

In 2017, Yusuke S. Hori et al. [21] found that FDP > 5 μg/mL was an independent risk factor for postoperative CSDH recurrence in 92 CSDH patients during postoperative follow-up, and the included CSDH patients were divided into the FDP > 5 μg/mL group and the FDP ≤ 5 μg/mL group. The overall recurrence rates after surgery were 27.3% and 10.2%, respectively, with the former having a recurrence rate 2.6 times higher than that of the latter. The recurrence rates one month after surgery were 15.2% and 3.4%, respectively, with the former having a recurrence rate approximately 4.5 times higher than that of the latter. There was no significant difference in the recurrence rates between the two groups for patients in the period of 31 to 90 days after surgery. The D-dimer findings of the current study significantly differed between the recurrence and nonrecurrence groups; the subsequent multifactorial binary logistic regression analysis indicated that D-dimer (OR = 0.053, 95% CI: 0.021–0.137, *p* < 0.001) was an independent risk factor for postoperative recurrence of CSDH; and the mechanism may be related to the localized D-dimer-induced coagulation and the inflammatory processes caused by D-dimer. Edlmann et al. [22] showed in their study that the pathophysiological mechanisms of CSDH include inflammation and proinflammatory cytokines such as interleukins 1, 6, and 8, and clinical studies have shown that the use of steroids is effective in the treatment of CSDH, that the elevation of serum FDP and D-dimer triggers systemic and local inflammatory responses and that active local inflammation promotes hematoma expansion. Active local inflammation can promote hematoma expansion and lead to recurrence of CSDH. Many studies have reported the relationships between D-dimer, FDP, and various inflammatory factors and CSDH.

It should be emphasized that plasma D-dimer elevation represents a nonspecific laboratory finding that can be triggered by diverse systemic pathological conditions, including trauma, bacterial infection, deep-vein thrombosis, and malignant tumors. In the present retrospective study, we excluded patients suffering from severe underlying diseases according to our predefined exclusion criteria, which partially mitigated these potential confounding effects; nevertheless, multivariate logistic regression still identified high preoperative D-dimer as an independent risk factor for postoperative CSDH recurrence. Pathophysiologically, elevated circulating D-dimer reflects persistent local hyperfibrinolytic and inflammatory activity within the subdural hematoma membrane: sustained fibrin deposition followed by fibrinolysis drives angiogenesis within the outer hematoma membrane, and these fragile newly formed microvessels are susceptible to repeated micro-oozing, thereby creating a self-amplifying vicious cycle through crosstalk between coagulation activation and local inflammation in which proinflammatory cytokines further boost fibrinolytic activity and consequently exacerbate micro-bleeding and hematoma re-accumulation. From a clinical perspective, preoperative D-dimer is an inexpensive and routinely accessible laboratory biomarker. Combined with our established nomogram, D-dimer contributes to individualized preoperative recurrence risk stratification. For patients with markedly elevated D-dimer who are stratified into the high-recurrence-risk group, clinicians may consider optimized surgical strategies such as neuroendoscopic membranectomy instead of simple burr-hole drainage, intensified postoperative imaging surveillance, or adjuvant anti-inflammatory interventions to reduce the risk of postoperative recurrence.

## 5. Conclusions

D-dimer is found to be a novel risk factor, and high levels of D-dimer predict recurrence in patients with CSDH. In the present study, we developed and externally validated a practical nomogram integrating seven readily available variables: age, hematoma volume on CT scan, hematoma location, cerebral atrophy, hematoma density pattern, surgical modality, and plasma D-dimer. This prediction model enables clinicians to quantitatively assess individual recurrence risk preoperatively, which facilitates risk-stratified clinical decision-making. For patients stratified into the high-recurrence-risk group, tailored perioperative strategies can be considered, such as selecting neuroendoscopic-assisted surgery instead of conventional burr-hole drainage, strengthening postoperative imaging surveillance, and implementing appropriate anti-inflammatory adjuvant interventions to reduce postoperative recurrence and complication rates. Nevertheless, this study has inherent limitations stemming from its retrospective single- and dual-center design. Further prospective multicenter studies are warranted to validate and refine this nomogram before its widespread clinical application.

## Figures and Tables

**Table 1:** Baseline characteristics of all patients in the training cohort and validation cohort.

Variables		Training Cohort (*n* = 632) N (%)	Validation Cohort (*n* = 206) N (%)	*p*-Value
Gender, *n* (%)	male	471 (74.5%)	151 (73.3%)	0.727
	female	161 (25.5%)	55 (26.7%)	
Age, y, *n* (%)	<70	371 (58.7%)	112 (54.4%)	0.536
	>80	95 (15%)	33 (16%)	
	70~80	166 (26.3%)	61 (29.6%)	
Smoke, *n* (%)	No	238 (37.7%)	79 (38.3%)	0.026
	Yes	394 (62.3%)	127 (61.7%)	
Drink, *n* (%)	No	474 (75%)	153 (74.3%)	0.834
	Yes	158 (25%)	53 (25.7%)	
History of hypertension, *n* (%)	Yes	461 (72.9%)	150 (72.8%)	0.971
	No	171 (27.1%)	56 (27.2%)	
History of diabetes, *n* (%)	Yes	189 (29.9%)	72 (35%)	0.012
	No	443 (70.1%)	134 (65%)	
Previous craniotomy, *n* (%)	No	564 (89.2%)	191 (92.7%)	0.147
	Yes	68 (10.8%)	15 (7.3%)	
Anticoagulation treatment, *n* (%)	Yes	159 (25.2%)	50 (24.3%)	0.798
	No	473 (74.8%)	156 (75.7%)	
Antiplatelet treatment, *n* (%)	No	439 (69.5%)	149 (72.3%)	0.435
	Yes	193 (30.5%)	57 (27.7%)	
History of head trauma, *n* (%)	Yes	337 (53.3%)	105 (51%)	0.008
	No	295 (46.7%)	101 (49%)	
CT hematoma size, cm^3^, *n* (%)	<100	491 (77.7%)	156 (75.7%)	0.819
	100~160	99 (15.7%)	36 (17.5%)	
	>160	42 (6.6%)	14 (6.8%)	
Midline shift, mm, *n* (%)	<0.5	399 (63.1%)	128 (62.1%)	0.797
	≥0.5	233 (36.9%)	78 (37.9%)	
Hematoma location, *n* (%)	unilateral	487 (77.1%)	150 (72.8%)	0.216
	bilateral	145 (22.9%)	56 (27.2%)	
Atrophy, *n* (%)	No	490 (77.5%)	164 (79.6%)	0.531
	Yes	142 (22.5%)	42 (20.4%)	
Density, *n* (%)	Membranous	81 (12.8%)	19 (9.2%)	0.258
	Separated	142 (22.5%)	58 (28.2%)	
	Mixed	275 (43.5%)	89 (43.2%)	
	Homogeneous	134 (21.2%)	40 (19.4%)	
GCS, *n* (%)	12~15	487 (77.1%)	152 (73.8%)	0.338
	5~11	145 (22.9%)	54 (26.2%)	
Surgical procedure, *n* (%)	Craniotomy	84 (13.3%)	27 (13.1%)	0.796
	Neuroendoscope	199 (31.5%)	70 (34%)	
	Burr hole craniostomy	349 (55.2%)	109 (52.9%)	
Platelet, *n* (%)	100~300	382 (60.4%)	126 (61.2%)	0.974
	>300	177 (28%)	56 (27.2%)	
	<100	73 (11.6%)	24 (11.7%)	
Albumin, *n* (%)	>40	423 (66.9%)	135 (65.5%)	0.736
	30~40	136 (21.5%)	43 (20.9%)	
	<30	73 (11.6%)	28 (13.6%)	
D-dimer, mg/L, *n* (%)	>2	45 (7.1%)	14 (6.8%)	0.819
	0.5~2	90 (14.2%)	33 (16%)	
	<0.5	497 (78.6%)	159 (77.2%)	
INR, *n* (%)	2~3	323 (51.1%)	104 (50.5%)	0.927
	<2	200 (31.6%)	68 (33%)	
	>3	109 (17.2%)	34 (16.5%)	

**Table 2:** The multivariate and univariate logistic regression analyses of chronic subdural hematoma.

Characteristics		No Recurrence	Recurrence	Total (N)	Univariate Analysis			Multivariate Analysis	
		N = 540	N = 92	N = 632	Odds Ratio (95% CI)	*p* Value	β	Odds Ratio (95% CI)	*p* Value
Age, y, *n* (%)	<70	329 (52.1%)	42 (6.6%)	371	Reference			Reference	
	>80	71 (11.2%)	24 (3.8%)	95	2.648 (1.508–4.651)	**<0.001**	0.9064	2.475 (1.078–5.682)	**0.033**
	70~80	140 (22.2%)	26 (4.1%)	166	1.455 (0.858–2.466)	0.164	0.4043	1.498 (0.724–3.101)	0.276
Smoke, *n* (%)	No	210 (33.2%)	28 (4.4%)	238	Reference				
	Yes	330 (52.2%)	64 (10.1%)	394	1.455 (0.903–2.343)	0.123			
Drink, *n* (%)	No	400 (63.3%)	74 (11.7%)	474	Reference				
	Yes	140 (22.2%)	18 (2.8%)	158	0.695 (0.401–1.204)	0.195			
History of hypertension, *n* (%)	Yes	387 (61.2%)	74 (11.7%)	461	Reference				
	No	153 (24.2%)	18 (2.8%)	171	0.615 (0.356–1.064)	0.082			
History of diabetes, *n* (%)	Yes	167 (26.4%)	22 (3.5%)	189	Reference				
	No	373 (59%)	70 (11.1%)	443	1.425 (0.853–2.379)	0.176			
Anticoagulation treatment, *n* (%)	Yes	140 (22.2%)	19 (3%)	159	Reference				
	No	400 (63.3%)	73 (11.6%)	473	1.345 (0.783–2.308)	0.283			
Antiplatelet treatment, *n* (%)	No	382 (60.4%)	57 (9%)	439	Reference				
	Yes	158 (25%)	35 (5.5%)	193	1.485 (0.937–2.351)	0.092			
History of head trauma, *n* (%)	Yes	297 (47%)	40 (6.3%)	337	Reference			Reference	
	No	243 (38.4%)	52 (8.2%)	295	1.589 (1.017–2.481)	**0.042**	0.5708	1.770 (0.946–3.312)	0.074
CT hematoma size, cm^3^, *n* (%)	<100	443 (70.1%)	48 (7.6%)	491	Reference			Reference	
	100~160	75 (11.9%)	24 (3.8%)	99	2.953 (1.708–5.107)	**<0.001**	1.4637	4.322 (1.951–9.574)	**<0.001**
	>160	22 (3.5%)	20 (3.2%)	42	8.390 (4.273–16.475)	**<0.001**	2.3978	11.000 (4.103–29.488)	**<0.001**
Hematoma location, *n* (%)	unilateral	443 (70.1%)	44 (7%)	487	Reference			Reference	
	bilateral	97 (15.3%)	48 (7.6%)	145	4.982 (3.132–7.926)	**<0.001**	1.4307	4.182 (2.172–8.051)	**<0.001**
Atrophy, *n* (%)	No	448 (70.9%)	42 (6.6%)	490	Reference			Reference	
	Yes	92 (14.6%)	50 (7.9%)	142	5.797 (3.632–9.253)	**<0.001**	1.8299	6.234 (3.214–12.091)	**<0.001**
Density, *n* (%)	Membranous	64 (10.1%)	17 (2.7%)	81	Reference			Reference	
	Separated	98 (15.5%)	44 (7%)	142	1.690 (0.889–3.213)	0.109	0.8368	2.309 (0.935–5.701)	0.070
	Mixed	259 (41%)	16 (2.5%)	275	0.233 (0.111–0.485)	**<0.001**	−1.0863	0.337 (0.128–0.886)	**0.027**
	Homogeneous	119 (18.8%)	15 (2.4%)	134	0.475 (0.222–1.013)	0.054	−0.9668	0.380 (0.124–1.171)	0.092
GCS, *n* (%)	12~15	422 (66.8%)	65 (10.3%)	487	Reference				
	5~11	118 (18.7%)	27 (4.3%)	145	1.486 (0.907–2.432)	0.116			
Surgical procedure, *n* (%)	Craniotomy	76 (12%)	8 (1.3%)	84	Reference			Reference	
	Neuroendoscope	189 (29.9%)	10 (1.6%)	199	0.503 (0.191–1.322)	0.163	−0.7235	0.485 (0.131–1.801)	0.280
	burr hole craniostomy	275 (43.5%)	74 (11.7%)	349	2.556 (1.181–5.534)	**0.017**	1.3931	4.027 (1.347–12.043)	**0.013**
Albumin, *n* (%)	>40	357 (56.5%)	66 (10.4%)	423	Reference				
	30~40	118 (18.7%)	18 (2.8%)	136	0.825 (0.471–1.446)	0.502			
	<30	65 (10.3%)	8 (1.3%)	73	0.666 (0.305–1.452)	0.307			
D-dimer, mg/L, *n* (%)	>2	22 (3.5%)	23 (3.6%)	45	Reference			Reference	
	0.5~2	59 (9.3%)	31 (4.9%)	90	0.503 (0.243–1.041)	0.064	−0.5812	0.559 (0.203–1.539)	0.261
	<0.5	459 (72.6%)	38 (6%)	497	0.079 (0.040–0.155)	**<0.001**	−2.9304	0.053 (0.021–0.137)	**<0.001**
INR, *n* (%)	2~3	280 (44.3%)	43 (6.8%)	323	Reference				
	<2	167 (26.4%)	33 (5.2%)	200	1.287 (0.786–2.105)	0.316			
	>3	93 (14.7%)	16 (2.5%)	109	1.120 (0.603–2.083)	0.720			

## Data Availability

All raw data used in this study were obtained from the public GEO database (GSE24265). No new data were generated in this study.
